# Population Genetic Structure of Listeria monocytogenes Strains as Determined by Pulsed-Field Gel Electrophoresis and Multilocus Sequence Typing

**DOI:** 10.1128/AEM.00583-16

**Published:** 2016-08-30

**Authors:** Clémentine Henri, Benjamin Félix, Laurent Guillier, Pimlapas Leekitcharoenphon, Damien Michelon, Jean-François Mariet, Frank M. Aarestrup, Michel-Yves Mistou, René S. Hendriksen, Sophie Roussel

**Affiliations:** a University Paris-Est, Anses, Maisons-Alfort Laboratory for Food Safety, Maisons-Alfort, France; b Technical University of Denmark, National Food Institute, WHO Collaborating Center for Antimicrobial Resistance in Foodborne Pathogens and European Union Reference Laboratory for Antimicrobial Resistance, Kongens Lyngby, Denmark; INRS–Institut Armand-Frappier

## Abstract

Listeria monocytogenes is a ubiquitous bacterium that may cause the foodborne illness listeriosis. Only a small amount of data about the population genetic structure of strains isolated from food is available. This study aimed to provide an accurate view of the L. monocytogenes food strain population in France. From 1999 to 2014, 1,894 L. monocytogenes strains were isolated from food at the French National Reference Laboratory for L. monocytogenes and classified according to the five risk food matrices defined by the European Food Safety Authority (EFSA). A total of 396 strains were selected on the basis of different pulsed-field gel electrophoresis (PFGE) clusters, serotypes, and strain origins and typed by multilocus sequence typing (MLST), and the MLST results were supplemented with MLST data available from Institut Pasteur, representing human and additional food strains from France. The distribution of sequence types (STs) was compared between food and clinical strains on a panel of 675 strains. High congruence between PFGE and MLST was found. Out of 73 PFGE clusters, the two most prevalent corresponded to ST9 and ST121. Using original statistical analysis, we demonstrated that (i) there was not a clear association between ST9 and ST121 and the food matrices, (ii) serotype IIc, ST8, and ST4 were associated with meat products, and (iii) ST13 was associated with dairy products. Of the two major STs, ST121 was the ST that included the fewest clinical strains, which might indicate lower virulence. This observation may be directly relevant for refining risk analysis models for the better management of food safety.

**IMPORTANCE** This study showed a very useful backward compatibility between PFGE and MLST for surveillance. The results enabled better understanding of the population structure of L. monocytogenes strains isolated from food and management of the health risks associated with L. monocytogenes food strains. Moreover, this work provided an accurate view of L. monocytogenes strain populations associated with specific food matrices. We clearly showed that some STs were associated with food matrices, such as meat, meat products, and dairy products. We opened the way to source attribution modeling in order to quantify the relative importance of the main food matrices.

## INTRODUCTION

*L*isteria monocytogenes is a ubiquitous Gram-positive bacterium that is responsible for listeriosis. This bacterium may cause severe symptoms, such as septicemia and meningitis, in the immunocompromised and elderly populations as well as in pregnant women, who may give birth to stillborn infants or severely infected newborns (1). In France, morbidity related to listeriosis is low, with an incidence of 5.6 cases per million inhabitants in 2013. However, the mortality related to listeriosis is high, leading to a fatal outcome in up to 20% to 30% (2). The most common transmission route in humans occurs via consumption of food contaminated during manufacturing, postprocessing, or storage.

L. monocytogenes has been divided into four lineages: I, II, III, and IV (3). Although 13 serotypes have been described, the majority of foodborne strains causing human infections belong to serotypes 4b, 1/2b (lineage I), 1/2a, and 1/2c (lineage II) (4). Molecular methods have been developed for use in the molecular surveillance of L. monocytogenes. Doumith et al. (5) described a multiplex PCR based on the presence or absence of four genetic markers, which cluster the strains into five molecular serogroups. This method has limited discriminatory power. Pulsed-field gel electrophoresis (PFGE) has been widely used for the molecular typing of L. monocytogenes. Although it is highly discriminatory, it is a time-consuming and labor-intensive method. Whole-genome sequencing (WGS) shows great promise for typing L. monocytogenes strains and has been used for the investigation of outbreaks (http://www.ssi.dk/English/News/News/2014/2014_08_listeria.aspx) (6). Multilocus sequence typing (MLST), which detects variations in the internal fragments of seven housekeeping genes (7), can also be deduced from WGS results. Each allele is assigned a number, the sequence type (ST) of a strain is determined by the combination of alleles, and clonal complexes (CCs) are designated clusters of STs that share at least six alleles.

In France, PFGE remains an invaluable tool for the routine surveillance of food and clinical strains (8). The French Agency for Food, Environmental and Occupational Health & Safety (Anses) is designated the French National Reference Laboratory (NRL) and the European Union Reference Laboratory (EURL) for L. monocytogenes. In collaboration with partners, it has established large collections of French and European food and clinical strains and molecular PFGE typing databases (9). In the literature, MLST studies have indicated that a few important clonal complexes account for the majority of listeriosis outbreaks and sporadic cases in France (7, 10, 11). At this time, knowledge of the molecular determinants leading to the prevalence of these clonal complexes in human infections remains limited. Regarding strains isolated from food, few studies are available on the genetic diversity of French strains. Most literature refers to PFGE typing studies, including strains isolated within the same specific food product types, such as cold smoked salmon (12), pork (13), or eggs (14). Only one study (15) compared the diversity between three different food matrices. Nevertheless, those investigations focused on a small panel of about 100 strains. To date, in France, no data are available on a large and diverse panel of strains.

The purpose of this study was to provide an overview of the population structure of L. monocytogenes strains isolated over the last 20 years in France. This should enable better management and understanding of food-related health risks. More specifically, the genetic diversity of L. monocytogenes strains originating from Anses was assessed by PFGE, and this allowed for the selection of a subset of strains for further analysis. This subset was chosen on the basis of different PFGE clusters, molecular serotypes, and strain origins. This panel, representing the genetic diversity of L. monocytogenes strains of food origin, was typed by MLST. The data were supplemented with MLST data available from Institut Pasteur, representing human and food strains from France. The distribution of sequence types was then compared between clinical and food strains.

## MATERIALS AND METHODS

### Description of the Anses PFGE database.

The Anses molecular database contains 1,894 L. monocytogenes strains collected over the last 20 years and centralizes detailed epidemiological information (sampling stage, context, source, food matrix, and food product) linked with genotypic (serotyping and PFGE) data for all of the strains.

The majority of the strains originated from diagnostic food laboratories and were isolated in different types of retailers and from various regions in France, covering a wide geographical area. They were collected between 1999 and 2014 based on self-checks or official sampling carried out by competent authorities (national control/monitoring programs and surveys). The database included 34 strains labeled TS (testing study) (16) (see Table S1 in the supplemental material) that were from the World Health Organization (WHO) international multicenter L. monocytogenes typing study. The database also contained strains from collaborative research projects with national and European partner laboratories, such as Ifip (French Pig and Pork Institute, France), INRA (French National Institute for Agricultural Research, France), PHE (Public Health England, United Kingdom), and SSI (Statens Serum Institute, Denmark). These projects allowed us to examine strains isolated from clinical cases, from animals, and from the natural environment. Finally, two fully sequenced reference strains, CLIP 80459 (17) and EGD-e (18), were also included in the database.

### Molecular serotyping.

All of the 1,894 L. monocytogenes strains were typed according to the Anses serotyping protocol, which is based on the amplification of the following target genes: *prs*, *lmo0737*, *lmo1118*, open reading frame 2110 (ORF2110), ORF2819, and *prfA* (species specific) described by Doumith et al. (5). The protocol clusters strains into five molecular serogroups, IIa (1/2a, 3a), IIb (1/2b, 3b, 7), IIc (1/2c, 3c), IVa (4a, 4c), and IVb (4b, 4ab, 4d, 4e), and the described variant profile of molecular serogroup IVb (IVb-v1) was characterized by the amplification of a supplementary *lmo0737* gene fragment (19).

### PFGE.

All of the 1,894 L. monocytogenes strains were typed and interpreted by PFGE using the protocol previously described in Félix et al. (20). For each of the strains, a combined PFGE pulsotype was defined based on the ApaI pulsotype/AscI pulsotype. The ApaI and AscI pulsotypes were considered different if there was at least one different band. Furthermore, each ApaI and AscI pulsotype was arbitrarily assigned to a pulsotype number (21). Pulsotype clustering was composed of profiles with 80% similarity (unweighted pair group method with arithmetic average [UPGMA]), with Dice's coefficient, tolerance, and optimization set at 1%. The pulsotype clustering and the number of strains in each cluster were established according to PFGE dendrogram clustering using a BioNumerics clustering script, and a numerical code was assigned to each cluster (21).

### MLST. (i) Selection of an MLST data panel representative of the diversity of L. monocytogenes in France.

From the Anses PFGE database, 15% of the strains from each PFGE cluster were selected for an MLST preliminary strain panel (396 strains) to reflect the diversity observed within the PFGE clusters and molecular serotypes. These data were supplemented with MLST results available in the articles of Institut Pasteur (7, 10, 11) from 279 strains. These strains were isolated in France between 1950 and 2006 and comprised 220 clinical strains, 17 food strains, 11 strains from the natural environment, 5 animal strains, and 25 strains of unknown origin. The entire MLST data set was subsequently compared with a panel of 675 strains in total, including 368 food strains and 241 clinical strains.

### (ii) MLST typing.

The Anses strain panel (396 strains) was typed by MLST. Of the 396 strains, DNA extractions for 298 were performed using an InstaGene Matrix kit from Bio-Rad (Bio-Rad Laboratories, CA, USA) to determine the MLST. MLST was achieved using two different sets of primers. Of the 298 strains, 130 were initially typed using the set of primers advised by the Listeria MLST database (http://bigsdb.pasteur.fr/listeria/primers_used.html), as the loci were too close to the landmark for most allelic sequences. The rest of the strains were typed by primers designed by Haase et al. (22) to ensure the full length of the sequences in each direction. For the PCR, initial denaturation at 94°C for 4 min was followed by 35 cycles of denaturation at 94°C for 30 s, annealing at 50°C for 30 s, and extension at 72°C for 2 min followed by a final extension step at 72°C for 10 min. The annealing temperature for the *lhkA* gene was set at 48°C instead of 50°C. PCR products were then purified and sequenced at EUROFINS (Eurofins GSC Lux SARL, Luxembourg).

The sequence type (ST) or allele profile was assigned to each strain based on the sequences of seven housekeeping genes described by Ragon et al. (7). Each new ST combination was sent for validation to the Listeria MLST database (http://bigsdb.pasteur.fr/listeria/primers_used.html).

The DNA of the remaining 98 strains was whole-genome sequenced using the Illumina HiSeq platform at the Wellcome Trust Centre for Human Genetics (Oxford, United Kingdom). DNA extraction was performed using an Invitrogen Easy-DNA genomic DNA (gDNA) purification kit (Invitrogen, Carlsbad, CA, USA). The raw reads were assembled using the Assembler pipeline (version 1.0) available from the Center for Genomic Epidemiology (CGE) (http://cge.cbs.dtu.dk/services/all.php), which is based on the Velvet algorithms for *de novo* short-read assembly. A complete list of genomic sequence data is available in Table S1 in the supplemental material. The assembled sequences were analyzed to identify the MLST for Listeria monocytogenes strains, and the minimum spanning tree (MST) was built using BioNumerics 7.5 software.

### Statistical analysis. (i) PFGE ID.

The ability of PFGE to discriminate unrelated strains was determined for each of the food matrices. The Simpson index of diversity (ID) (23) was calculated on the basis of the PFGE results obtained from epidemiologically unrelated strains in each food matrix.

### (ii) Association between food matrices and typing data.

The Gini coefficient, a measure of statistical dispersion, was calculated to evaluate association between food matrices and molecular serotypes/PFGE clusters/STs using the ineq package in R (24). This coefficient makes it possible to quantify molecular serotype/PFGE cluster or ST distribution among the strains in each food matrix. Values of the coefficient ranged from 0 (completely equal distribution such that each molecular serotype/PFGE cluster or ST is represented evenly among strains in the matrix) to 1 (completely unequal distribution where molecular serotype/PFGE cluster or ST represents all of the strains in the matrix). A Gini coefficient that is smaller than 0.4 indicates no association between molecular serotype/PFGE cluster or ST and food type matrices, values between 0.4 and 0.6 indicate moderate association, and values greater than 0.6 reveal unequal dispersion of molecular serotype/PFGE cluster or ST within food matrices.

### (iii) Congruence between MLST and PFGE.

PFGE data were not available for the 279 strains from Institut Pasteur. This is why MLST and PFGE data were compared only to the MLST subset from Anses (396 strains). Congruence was assessed using the adjusted Rand coefficient (25, 26). This coefficient (i) indicates the probability that a pair of strains which are assigned to the same type by one typing method are identically typed by another method, (ii) indicates the probability that a pair of strains which are assigned to two types by one typing method are differently typed by another method, and (iii) corrects the typing concordance for chance agreement, avoiding the overestimation of congruence between typing methods.

For a more detailed comparison, the adjusted Wallace (AW) coefficient (25) was assessed. The AW coefficient indicates the probability that pairs of strains which are assigned to the same type by one typing method are identically typed by another method and corrects the typing concordance for chance agreement. The AW coefficient is directional, i.e., given a standard method, it indicates the probability of two strains having the same type by the standard method also sharing the same type by the compared method. Statistical analyses were performed using a previously developed script (25, 26). Ninety-five percent confidence intervals were also calculated for the two coefficients.

## RESULTS

### Distribution of 1,894 strains according to origin and molecular serotype.

The 1,894 strains had four different origins: food and food processing environments (FPEs), humans, animals, and natural environments (see Table S2 in the supplemental material). The panel included a very large proportion of strains from food and FPEs (*n* = 1,698) (Table 1). The most prevalent food matrices were meat and meat products (*n* = 687), milk and milk products (*n* = 263), and fish and fishery products (*n* = 199) (Table 1).

**TABLE 1:** Distribution of the 1,698 food strains according to molecular serotype and food matrix

Lineage	Molecular serotype	No. of strains in the total panel	No. (%) of food and FPE strains	No. (%) of strains according to food matrix						FPE
				Meat and meat products	Milk and milk products	Fish and fishery products	Processed food products combining several foods	Fruits, vegetables, cereals, and herbs	Undetermined food matrices	
II	IIa	998	939 (55.3)	346 (36.8)	144 (15.3)	135 (14.4)	109 (11.6)	36 (3,8)	50 (5,3)	118 (12,6)
	IIc	260	247 (14,5)	159 (64,4)	9 (3,6)	10 (4,0)	22 (8,9)	3 (1,2)	11 (4,5)	33 (13,4)
I	IIb	212	179 (10.5)	64 (35.8)	33 (18.4)	26 (14.5)	19 (10.6)	4 (2.2)	9 (5.0)	24 (13.4)
	IVb	416	330 (19.4)	116 (35.2)	77 (23.3)	28 (8.5)	37 (11.2)	13 (3.9)	26 (7.9)	34 (10.3)
	IVb-variant	4	3 (0.2)	2 (66.7)				1 (33.3)		
III	IVa	4								
Total		1,894	1,698 (89.7)	687 (40.5)	263 (15.5)	199 (11.7)	187 (11.0)	57 (3.4)	96 (5.7)	209 (12.3)

**FIG 1:**
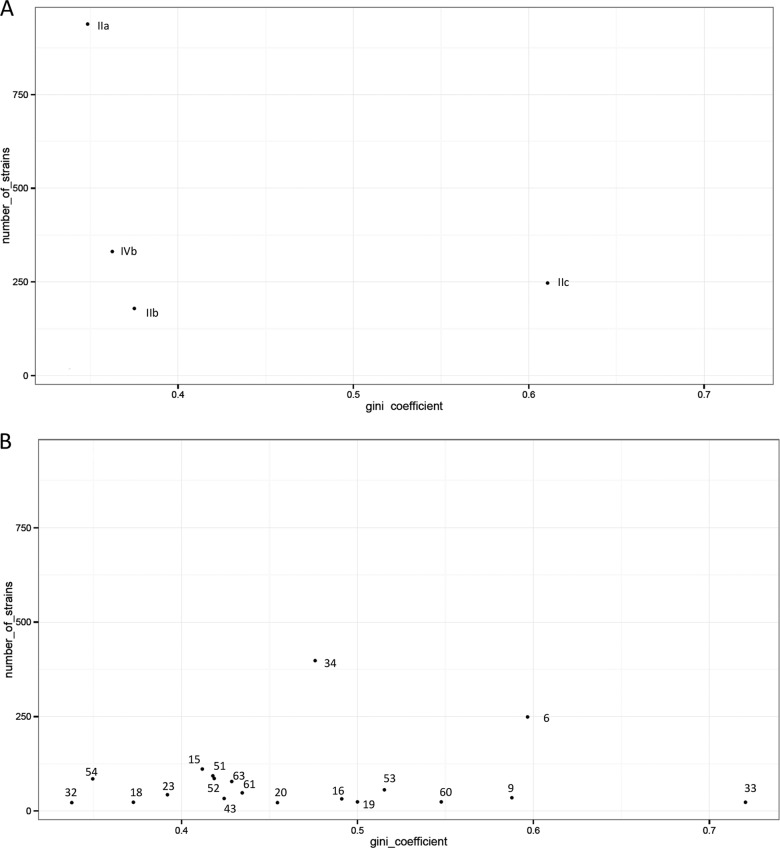
Gini coefficient values calculated on the panel of 1,698 L. monocytogenes food and food processing environment strains between food matrices and molecular serotypes (A) and PFGE clusters (B). Vertical scale indicates the number of strains in each molecular serotype (A) and PFGE cluster (B). Each number represents a PFGE cluster. Values of the Gini coefficient ranged from 0 (completely equal distribution, such that each serotype/cluster is represented evenly among strains in the matrix) to 1 (completely unequal distribution where one serotype/cluster represents all of the strains in the matrix). A Gini coefficient that is smaller than 0.4 indicates no association between serotype/profile and food type matrices, values between 0.4 and 0.6 indicate moderate association, and values greater than 0.6 reveal unequal dispersion of serotype/cluster within food matrices.

Of 1,698 food and FPE strains, the molecular serotype IIa was predominant in all food matrices, representing 55% of the panel. However, the potential of L. monocytogenes to contaminate food is not restricted to this molecular serotype, as the second most abundant is IVb (19%) (Table 1). Gini coefficient values calculated on the panel of 1,698 strains indicated that the three molecular serotypes IIa, IIb, and IVb were not associated with a particular food matrix and that, nevertheless, molecular serotype IIc was associated with the meat and meat products matrix (Fig. 1-A).

### Genetic diversity of the 1,894 strains tested by PFGE.

The 1,894 strains were divided into 73 PFGE clusters (see Table S3 in the supplemental material), with 19 main clusters containing at least 20 strains (Table 2). However, the repartition of the PFGE clusters was heterogeneous. We observed that 54 clusters contained fewer than 20 strains and represented less than 13% of the panel, while two clusters, 34 and 6, were considered to be of high abundance as they represented 21.2% and 13.8% of the panel, respectively (Table 2; see also Table S3).

**TABLE 2:** Distribution of the 19 main PFGE clusters according to food matrix

PFGE group no.	No. of strains in the total panel	No. (%) of food and FPE strains	No. (%) of strains according to food matrix						FPE
			Meat and meat products	Milk and milk products	Fish and fishery products	Processed food products combining several foods	Fruits, vegetables, cereals, and herbs	Undetermined food matrices	
34	401	398 (99.3)	168 (42.2)	9 (2.3)	71 (17.9)	72 (18.1)	11 (2.8)	9 (2.3)	58 (14.6)
6	262	249 (95.0)	158 (63.5)	11 (4.4)	7 (2.8)	22 (8.8)	3 (1.2)	19 (7.6)	29 (11.6)
15	128	111 (86.7)	59 (53.2)	10 (9.0)	8 (7.2)	9 (8.1)	9 (8.1)	7 (6.3)	9 (8.1)
51	121	93 (76.9)	38 (40.9)	5 (5.4)	16 (17.2)	13 (14.0)	2 (2.1)	12 (12.9)	7 (7.5)
54	117	85 (72.6)	28 (32.9)	21 (24.7)	6 (7.1)	10 (11.8)	4 (4.7)	8 (9.4)	8 (9.4)
52	92	78 (84.8)	26 (33.3)	26 (33.3)	3 (3.8)	7 (9.0)	4 (5.1)	4 (5.1)	8 (10.3)
63	92	86 (93.5)	37 (43.0)	9 (10.5)	8 (9.3)	10 (11.6)	3 (3.5)	4 (4.7)	15 (17.4)
53	65	56 (86.2)	16 (28.6)	21 (37.5)		6 (10.7)	2 (3.6)	1 (1.8)	10 (17.9)
61	61	48 (78.7)	18 (37.5)	10 (20.8)	4 (8.3)	7 (14.6)		2 (4.2)	7 (14.6)
23	47	43 (91.5)	14 (32.6)	4 (9.3)	2 (4.7)	5 (11.6)	6 (13.9)	1 (2.3)	11 (25.6)
9	39	35 (89.7)	12 (34.3)	15 (42.9)		1 (2.8)	4 (11.4)		3 (8.6)
43	36	33 (91.7)	12 (36.4)	6 (18.2)	7 (21.2)	3 (9.1)		3 (9.1)	2 (6.6)
60	34	24 (70.6)	4 (16.7)	6 (25)	11 (45.8)	1 (4.2)	1 (4.2)		1 (4.1)
16	33	32 (97.0)	8 (25)	1 (3.1)	13 (40.6)	4 (12.5)		4 (12.5)	2 (6.25)
18	31	23 (74.2)	3 (13.0)	5 (21.7)	5 (21.7)	1 (4.3)	1 (4.3)	1 (4.3)	7 (30.4)
19	24	24 (100)	9 (37.5)	5 (20.8)	5 (20.8)			5 (20.8)	
20	23	22 (95.7)	7 (31.8)	3 (13.6)	3 (13.6)	1 (4.5)		7 (31.8)	1 (4.5)
32	23	22 (95.7)	2 (9.1)	6 (27.3)	5 (22.7)	2 (9.1)	1 (4.5)	1 (4.5)	5 (22.7)
33	23	23 (100)	2 (8.7)	16 (69.6)		1 (4.3)			4 (17.4)
35	23	18 (78.3)	7 (38.9)	2 (11.1)	5 (27.8)	2 (11.1)			2 (11.1)
Subtotal 19 clusters	1,652	1,485	621	189	174	175	51	88	187
Others clusters remaining	242	213	66	74	25	12	6	8	22
Strain total	1,894	1,698	687	263	199	187	57	96	209

We noted that cluster 34 included 401 strains and contained mainly food and FPE strains (*n* = 398) (Table 2). Except for two strains of serotype IIa and IIc, all of the PFGE clusters were molecular serotype specific (see Table S4 in the supplemental material).

The ability of PFGE to discriminate unrelated strains within the same matrix was calculated by the Simpson's index of diversity (ID). The IDs were similar between the different food matrices and were high, ranging from 0.987 to 0.997 (see Table S5 in the supplemental material), showing that each food matrix contained many clusters and suggesting a great diversity of L. monocytogenes strains in the five food matrices.

Nonetheless, we were also interested to assess whether a PFGE cluster could be associated with a food matrix. Figure 1B represented the Gini coefficient for the 19 main PFGE clusters. These PFGE clusters represented more than 80% of our panel of food and FPE strains (Table 2). With values of the Gini coefficient below 0.4, four PFGE clusters were uniformly distributed in the five different food matrices (Fig. 1B). This means that these four PFGE clusters were not associated with a particular food matrix. However, one cluster was associated with a particular food matrix: cluster 33 was associated with milk and milk products (Fig. 1B and Table 2).

### MLST and congruence between STs and PFGE clusters.

From the Anses PFGE database, we selected a preliminary strain panel (396 strains) to reflect the diversity observed within the PFGE clusters, molecular serotypes, the origins of the strains, and food matrices. These strains were then typed by MLST. The 396 strains were distributed into 73 different STs, including 34 singletons, 28 STs that contained between two and nine strains, and 11 major STs containing at least 10 strains (ST121, ST9, ST1, ST2, ST5, ST31, ST8, ST6, ST4, ST13, and ST59) (see Table S6 in the supplemental material). Seven new STs (ST769, ST770, ST771, ST772, ST773, ST774, and ST775) were also observed (see Table S6 in the supplemental material).

Quantitative determination of concordance of the two methods was calculated based on those 396 strains. Congruence between the two methods was found to be high (adjusted Rand index of 0.843), with a confidence interval (CI) between 0.788 and 0.900. The AW PFGE to MLST was found to be 0.853 (CI of between 0.798 and 0.907), indicating that partitions defined by MLST may have been predicted from the results of PFGE clusters at 80% similarity with 85% accuracy.

The STs obtained and PFGE clusters were compared for this panel of 396 Anses strains (see Table S6 in the supplemental material). One of the two prevalent STs, ST9, corresponded to PFGE cluster 6 for 40 of the 45 strains typed by MLST. Of 71 strains included in the other major ST, ST121, 62 strains displayed the same PFGE cluster, cluster 34 (see Table S6 in the supplemental material).

### Genetic diversity compared between food, FPE, and clinical strains by MLST.

**FIG 2:**
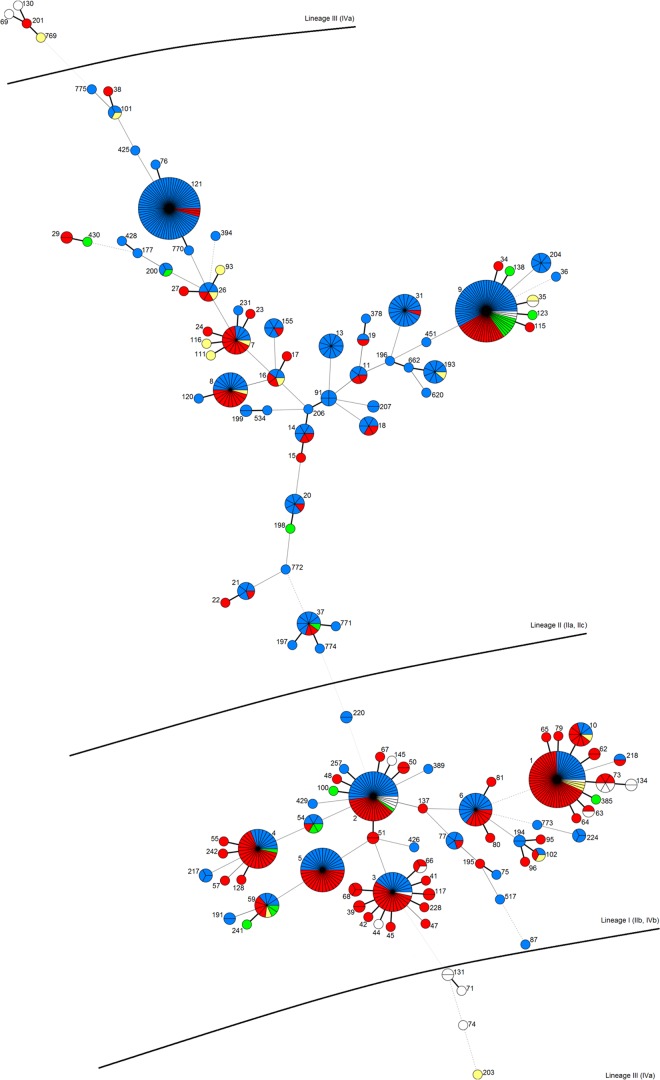
Minimum spanning tree of 675 L. monocytogenes strains according to their ST. Each circle represents a ST, and ST number is indicated beside the circle. Circle size is proportional to the number of isolates. Each slice in the circle represents one strain. The distance between circles represents genetic divergence. Thick solid lines represent one allele difference, thin solid lines represent two alleles, dashed lines represent three alleles, dotted lines represent four alleles, and, finally, the absence of a line means more than four alleles. Strain origins are indicated by color code. Blue, strains isolated from food; red, strains from clinical cases; green, strains from the natural environment; yellow, strains from animals.

The MLST data from the Anses strain panel were compared with the MLST results available in the articles of Institut Pasteur from 279 strains. The whole MLST data set included a total panel of 675 strains (including 368 food strains and 241 clinical strains) (see Table S7 in the supplemental material). The 675 strains were separated into 130 distinct STs and 46 CCs (see Table S7) correlating with L. monocytogenes genetic lineages I, II, and III (Fig. 2). In addition, the two reference strains complied with the expected genetic lineages. We observed that the four strains ST69, ST130, ST201, and ST769, belonging to lineage III, were split from four other strains, ST131, ST74, ST71, and ST203, of the same lineage (Fig. 2).

Among the 130 STs, we observed 77 singletons, 38 STs containing between two and nine strains, and 15 main STs containing more than 10 strains. Those 15 STs included clinical and food/FPE strains. The two major STs were ST9 (72 strains including 19 clinical strains) and ST121 (71 strains including three clinical strains). We mentioned that ST121 contained the fewest clinical strains (Fig. 2; see also Table S7 in the supplemental material).

**FIG 3:**
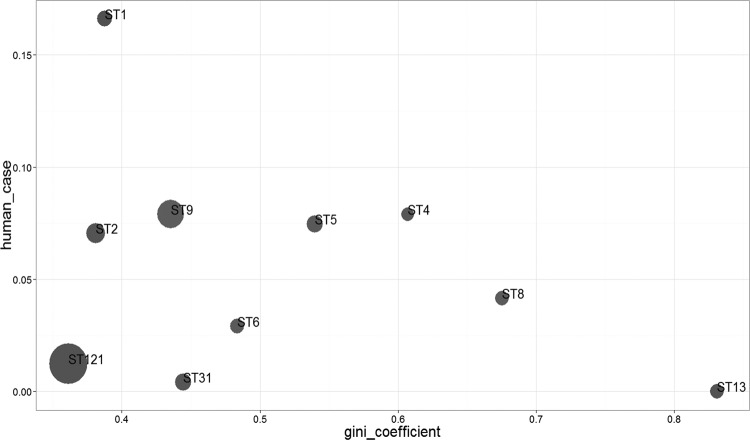
Gini coefficient values calculated on the food strains of the 675-strain panel between food matrices and the STs. The vertical scale indicates the percentage of human strains within the same ST. The size of the circle is proportional to the relative number of food strains in each ST. Values of the Gini coefficient ranged from 0 (completely equal distribution, such that each ST is represented evenly among strains in the matrix) to 1 (completely unequal distribution where one ST represents all the strains in the matrix). A Gini coefficient that is smaller than 0.4 indicates no association between ST and food matrices, values between 0.4 and 0.6 indicate moderate association, and values greater than 0.6 reveal unequal dispersion of ST within food matrices.

To quantify this potential preferential ST association, we performed a statistical analysis. Regarding the food matrices, Fig. 3 represented the Gini coefficients for the 10 STs that included at least 10 strains isolated from food products. Three STs were associated with food matrices, ST13 with dairy products and ST8 and ST4 with meat products. With Gini coefficient values below 0.4, three STs, ST121, ST2, and ST1, were uniformly distributed in the five different food matrices (Fig. 3).

While ST13 did not include any human strains, ST1 included more than 15% of human strains. ST4 and ST8 included 7% and 4% of human strains, respectively, and ST121 included 2% of human strains.

STs with low Gini coefficients contributed the most to consumer exposure to L. monocytogenes, as these STs were found in almost every food categories. Yet, high exposure for some STs was not associated with a strong contribution in human listeriosis cases (Fig. 3). Strains belonging to ST121, while being the most present ST in number and diversity of food matrices, represented less than 1% of clinical cases (Fig. 3). This indicated that implication in listeriosis cases is underpinned by other factors than exposure, most likely virulence factors.

Consequently, the high contributions to listeriosis cases of ST1, ST2, ST9, ST4, and ST5 may be explained by different causes. For strains of ST2 and ST9, their presence in numbers in almost all food categories may explain their large contribution to listeriosis cases. Strains in ST1, which represent lower exposure in number than ST2 or ST9, contribute more than these STs to clinical cases, indicating the higher virulence of these strains. For ST4 and ST5, their relatively high Gini coefficients indicate a lower exposure to these STs whereas their contribution to clinical cases is important. As for ST1, this ST may present a higher virulence than other STs.

## DISCUSSION

This study describes the structure of the L. monocytogenes strain population from the different food production sectors in France. Of the total strain panel (1,894), more than half (52.4%) were of molecular serotype IIa. This result was consistent with previous studies (27, 28). The most frequent genetic PFGE groups observed here were lineage II groups, corresponding to ST9 and ST121. Previously, MLST analysis performed on smaller collections of food strains in Italy, Spain, and Switzerland also showed the predominance of ST9 and ST121 (27–29, 30).

Here, we classified the strains according to the five main food matrices defined by the European Food Safety Authority (EFSA). This classification takes into account the risk food matrix known for L. monocytogenes in Europe (9). The present work provides data on the distributions of genetic groups, such as PFGE clusters or STs, in the different food matrices. Through the combination of three different typing methods associated with original statistical analysis, this study contributes to an accurate view of the L. monocytogenes population associated with specific food matrices. Indeed, we clearly showed that (i) there was no association between the three sequence types ST121, ST2, and ST1 and the food matrices, (ii) molecular serotype IIc, ST8, and ST4 were associated with meat and meat products, and (iii) ST13 was associated with milk and milk products. These results suggest that the presence of genetic markers associated with these two risk food matrices may be identified by future comparative genomic analysis. This work also opens the way to source attribution modeling in order to quantify the relative importance of the main food matrices.

To compare the genetic structures of L. monocytogenes strains isolated from food with those of clinical strains, we built a large and balanced strain data set. The analysis of the structures of 675 L. monocytogenes strains from France, including clinical and food strains, revealed two prevalent STs: ST9 and ST121. In the present study, ST9 corresponded to both clinical and food strains. In contrast, out of the main STs obtained here, ST121 included the least clinical strains. We then suggest that ST121 strains may have genetic characteristics that drive them to be successful in food and the food processing environment but that they are less pathogenic for humans. Similar conclusions regarding ST9 and ST121 were recently drawn from MLST analysis performed on a large collection of food and clinical isolates (4).

ST121 strains have previously been isolated from food and food processing facilities over several years in processing plants in Denmark (31), Austria, and Belgium (32). Holch et al. (31) investigated two ST121 strains isolated from two different Danish fish processors using WGS. The genomic and proteomic comparisons indicated that the two strains were almost identical, with a predicted protein homology of 99.94%, differing at only two proteins. These strains were distinguished by two genome deletions: one of 2,472 bp that typically contained the gene *inlF* and the other of 3,017 bp that included three genes potentially related to bacteriocin production and transport. By PCR, Hein et al. (32) showed that an ST121 strain had a deletion in the survival islet SSI-1. Transcriptional analysis demonstrated that the islet genes are internally regulated by *Lmo0445*, suggesting that this regulator may contribute to the capacity of L. monocytogenes to respond and adapt to the various environmental conditions encountered either in foods or within the host (33). Except these studies, few data are available on the genetic makeup of the ST121 strains. Investigations comparing the genomes of ST121 strains with those of strains from ST lineage II, which is known as being responsible for listeriosis, may reveal genetic features and allow us to investigate the genetic basis of the hypovirulence potential of L. monocytogenes food strains.

STs with low Gini coefficients, such as ST121, ST2, and ST1, were found in almost every food category. A hypothesis is that these STs may contribute the most to the consumer exposure to L. monocytogenes. For ST1 and ST2 strains, it may explain their large contribution to listeriosis cases. However, strains belonging to ST121, while being the most present ST in number and diversity of food matrices, represented less than 1% of clinical case. This indicated that, for ST121 strains, implication in listeriosis cases may be underpinned by other factors than exposure, most likely virulence factors. Consequently, the high contributions to listeriosis cases of ST1 may be explained by other causes, i.e., strains in ST1 may present a higher virulence than those in other STs.

The virulence is known to differ greatly between L. monocytogenes strains (34, 35). Probability of listeriosis associated with the ingestion of a few cells can be 1,000 times greater with one strain than another (35). In the same way, in quantitative exposure assessment of L. monocytogenes, the variability of behavior in the food chain is usually incorporated (36) but is not related to subtypes. Quantitative risk assessments would certainly reduce the uncertainty associated with risk estimates by taking into account subtyping in exposure assessment and dose response.

Regarding the typing methods, in France and Europe, many national reference laboratories and national public health reference laboratories (NPHRLs) continue to use PFGE for routine surveillance and outbreak investigations (8, 37). Given the well-known drawbacks of PFGE, MLST can be used as a first-line method for routine surveillance of food and clinical L. monocytogenes strains. In this study, high congruence values between PFGE and MLST were obtained on the panel of 396 strains tested by the two methods. The obtained data make mapping possible between the PFGE clusters, clonal complexes, and STs. This correspondence is very useful for the centralized European molecular typing databases maintained by the EURL for L. monocytogenes (9). The future European Centre for Disease Prevention and Control (ECDC)-EFSA database (38) should also benefit from this correspondence.

Although the MLST scheme used here, based on seven housekeeping genes, has been found to be very useful for phylogeny and population structure investigations (7), one of the limitations is the low number of genes taken into account. For a more detailed analysis of the population structure, we suggest using other methods, such as WGS based on single nucleotide variants (single nucleotide polymorphisms [SNPs]) or a scheme based on genome-wide gene-by-gene comparison called core genome MLST (cgMLST) (39). Core genome MLST extends the concept of MLST to core genome sequences and should allow us to identify all genes in the strain genome to determine allelic variants for each gene and record the absence or presence of accessory genes (40).

In conclusion, this study showed a very useful backward compatibility between PFGE and MLST for surveillance. The population structure of the strains isolated in France revealed two frequently observed sequence types: ST9 and ST121. There was not a clear association between these two STs and the food matrices. Interestingly, ST121 included the fewest clinical strains, indicating lower virulence. Further comparative genomics will be needed to characterize regions that may explain the observed differences in the hypovirulence of strains. This work will contribute to better understanding of the health risks associated with L. monocytogenes.

## Supplementary Material

Supplemental material
