# Supplementary material for: Population Genetic Structure of Listeria monocytogenes Strains as Determined by Pulsed-Field Gel Electrophoresis and Multilocus Sequence Typing
Source: Appl Environ Microbiol. 2016 Aug 30;82(18):5720–8. doi: 10.1128/AEM.00583-16 (PMC5007763; doi:10.1128/AEM.00583-16)
Supplement: Supplemental material [file supp_82_18_5720__index.html]

Supplemental material 

# Population Genetic Structure of Listeria monocytogenes Strains as Determined by Pulsed-Field Gel Electrophoresis and Multilocus Sequence Typing

## Supplemental material

- Supplemental file 1 -

  Testing study strains (Table S1), distribution of strains by molecular serotype and origins (Table S2) and in the PFGE clusters according to origins and food matrices (Table S3) and molecular serotypes (Table S4), PFGE index of diversity in each food matrix (Table S5), contingence table (Table S6), and distribution of strains within the STs according to origin and food matrix (Table S7).

  XLSX, 68K
